# Recent advances in 3D printing of biomaterials

**DOI:** 10.1186/s13036-015-0001-4

**Published:** 2015-03-01

**Authors:** Helena N Chia, Benjamin M Wu

**Affiliations:** Department of Bioengineering, Henry Samueli School of Engineering, University of California, 5121 Engineering V, Los Angeles, CA 90095 USA; Department of Materials Science and Engineering, Henry Samueli School of Engineering, University of California, Los Angeles, CA 90095 USA; Division of Advanced Prosthodontics, School of Dentistry, University of California, Los Angeles, CA 90095 USA; Department of Orthopedic Surgery, School of Medicine, University of California, Los Angeles, CA 90095 USA

**Keywords:** 3D Printing, Fused deposition modeling, Selective laser sintering, Stereolithography, Computer-aided tissue engineering, 3D plotting, Bioprinting

## Abstract

3D Printing promises to produce complex biomedical devices according to computer design using patient-specific anatomical data. Since its initial use as pre-surgical visualization models and tooling molds, 3D Printing has slowly evolved to create one-of-a-kind devices, implants, scaffolds for tissue engineering, diagnostic platforms, and drug delivery systems. Fueled by the recent explosion in public interest and access to affordable printers, there is renewed interest to combine stem cells with custom 3D scaffolds for personalized regenerative medicine. Before 3D Printing can be used routinely for the regeneration of complex tissues (e.g. bone, cartilage, muscles, vessels, nerves in the craniomaxillofacial complex), and complex organs with intricate 3D microarchitecture (e.g. liver, lymphoid organs), several technological limitations must be addressed. In this review, the major materials and technology advances within the last five years for each of the common 3D Printing technologies (Three Dimensional Printing, Fused Deposition Modeling, Selective Laser Sintering, Stereolithography, and 3D Plotting/Direct-Write/Bioprinting) are described. Examples are highlighted to illustrate progress of each technology in tissue engineering, and key limitations are identified to motivate future research and advance this fascinating field of advanced manufacturing.

## Introduction

The ability to design and fabricate complex, 3D biomedical devices is critical in tissue engineering. Applications for 3D biomedical devices are restoration of 3D anatomic defects, the reconstruction of complex organs with intricate 3D microarchitecture (e.g. liver, lymphoid organs), and scaffolds for stem cell differentiation. An example of a need is anatomic defects in the craniomaxillofacial complex caused by cancer, trauma, and congenital defects. Proper restoration of these defects requires functional nerves, vessels, muscles, ligaments, cartilage, bone, lymph nodes and glands.

In recent years, various approaches based on tissue engineering principles have been explored to regenerate other functional tissues that are relevant to maxillofacial tissue regeneration. In tissue engineering, scaffolds are critical to provide structure for cell infiltration and proliferation, space for extracellular matrix generation and remodeling, biochemical cues to direct cell behavior, and physical connections for injured tissue. When making scaffolds, design of the architecture on the macro, micro, and nano level is important for structural, nutrient transport, and cell-matrix interaction conditions [1-3]. The macroarchitecture is the overall shape of the device which can be complex (e.g. patient and organ specificity, anatomical features). The microarchitecture reflects the tissue architecture (e.g. pore size, shape, porosity, spatial distribution, and pore interconnection). The nanoarchitecture is surface modification (e.g. biomolecule attachment for cell adhesion, proliferation, and differentiation).

Although an ideal scaffold will account for all these factors, challenges still exist with biomaterial selection and 3D shape specificity. Biomaterials commonly used are polymers (synthetic and natural), ceramics, and metals. Each biomaterial has specific material and mechanical properties, processing methods, chemical properties, cell-material interactions, and FDA approval. Common fabrication methods to produce porosity and a range of pores size are gas foaming, solvent casting with particle leaching, freeze-drying, and eletrospinning. While the microarchitecture in these methods is well-controlled and understood, the ability to control macroarchitecture with these methods is limited to 3D shapes and geometries determined by molds and manual processing. The ability to incorporate internal architecture or curved channels is also limited when using these methods.

Solid free form fabrication (SFF) has allowed for the design and fabrication of complex 3D structures which can be patient specific. The integration of computer aided design, advanced imaging techniques (i.e. magnetic resonance imaging and computer tomography), and rapid prototyping has advanced fabrication of objects with both macro and microarchitecture control. In addition, patient specific imaging can be used to customize builds for individuals [4,5]. A type of rapid prototyping, SFF offers a method to control both the micro and macroarchitecture to create complex biomedical devices. Most surface modifications can be completed in post-processing. While conventional material processing techniques can be highly effective in scaffold engineering, SFF technologies offer exciting opportunities for tissue engineering of highly complex maxillofacial tissues. However, each technology has its limitations. The selection of the fabrication technique depends upon the materials of interest, machine limitations, and the specific requirements of the final scaffold.

The term “3D Printing” should be clarified to prevent confusion in this review article. Currently in literature and mainstream media, the term “3D Printing” is being used to refer to all SFF technologies (e.g. fused deposition modeling, selective laser sintering, etc.). In this review, the term will be used in two ways: to generally refer to all SFF technologies and to refer to the liquid binder-based inkjet technology which is described in detail below. The use of the term will be clear in the different sections.

The state of the art 3D Printing, especially for the production of implantable biomedical devices, is severely limited by printable materials. Therefore in most cases, alternative material processing methods are required to work with materials that are not easily printed. In cases where materials can be printed, 3D Printing is particularly advantages for one-of-a-kind, customized complex devices that are not cost effective in conventional manufacturing methods such as injection molding.

While industrial 3D printers have reached extremely high resolution in the past few years, the advancements in machine capability have not translated to the use with biomaterials. Industrial 3D printers can now reach extremely small build layers such as 16 μm layer thickness for SLA (Polyjet, Stratasys), 178 μm layer thickness for FDM (Fortus 900mc, Stratasys), 80 μm layer thickness for SLS (sPro 230HS, 3D Systems) and 75 μm resolution for SLA (3D Systems). These systems unfortunately are not optimized for biomaterials of interest for *in vitro* and *in vivo* studies and advances are still being made to improve SFF methods for biomaterials.

The cost of each of these technologies is currently difficult to compare since many advances are based on home-made setups or modification of commercial machines by creative engineers. Actual cost will be easier to compare when the materials become available for large scale adaptation for industrial 3D printers. That stage will also determine the ease of use for both printing and post-processing. Even with current modeling materials, most printers require some type of sacrificial support materials that require careful removal

SFF methods, particularly FDM, have recently exploded in popularity and gone viral. Machines are being developed specifically for home, school, and small business use with much lower price points and less complexity than industrial grade machines. In addition, low-cost consumer 3D scanners and free CAD software has allowed those interested in SFF to design and fabricate parts themselves at home. While these technologies were previously mainly limited to academia and industry, SFF has burst into mainstream use and many more people now understand the capability of the technologies.

This review focuses on advanced 3D Printing technologies that are being used to fabricate tissue engineering scaffolds, with emphasis on their ability of these manufacturing technologies to pattern cells and multiple materials along complex 3D gradients. Many of these technologies are already used for making patient specific models for pre-surgical planning, surgical templates and prosthesis fabrication. Some already gained FDA clearance for implantable devices. In particular, work done in the last five years will be highlighted to show the progression of the field.

## 3D printing of tissue engineering scaffolds

Most SFF methods build 3D biomedical devices in a layer-by-layer process. The general SFF process involves 1) creating a 3D computer model (can be generated from medical imaging data such as CT scans or X-rays) 2) slicing the 3D computer model into a build file of 2D images with software, 3) fabricating the build by a computer-controlled layer-by-layer process, and 4) finishing with any post processing such as surface modification for nanoarchitecture. Complicated three-dimensional features such as internal voids, cantilevers, undercuts, and narrow tortuous paths are simply reduced to a stack of common two-dimensional features such as circles, lines, and points. Exempted from tooling path restrictions, these additive technologies offer much higher levels in shape complexity. Although these SFF technologies were developed primarily for industrial applications, their flexibility in creating complex three-dimensional shapes make SFF technologies attractive candidates for biomedical engineering. Various SFF techniques were introduced to build objects with controlled macroarchitecture as well as microstructures with biomedical and tissue engineering applications. The freedom in form, combined with the appropriate material deposition technology offer control over the tissue engineering triad by simultaneously directing the spatial distribution of cells, signals, and scaffolding substrates during fabrication. Furthermore, these technologies allow integration between digitized medical imaging data with computer-aided-design models [5,6]. The integration of SFF technologies with patient-specific medical imaging data enables the aseptic manufacturing of tissue engineering grafts that match precisely to a patient’s contours can be produced by. These technologies enable the fabrication of multi-functional scaffolds that meet the structural, mechanical, and nutritional requirements based on optimized models [7].

For this review, a brief overview of five popular SFF technologies will be described, and examples of tissue engineering applications are provided. For each technology, recent advances in machine capability and printable biomaterials will be reviewed.

### Three dimensional printing

#### Technology description and application

**Figure 1:**
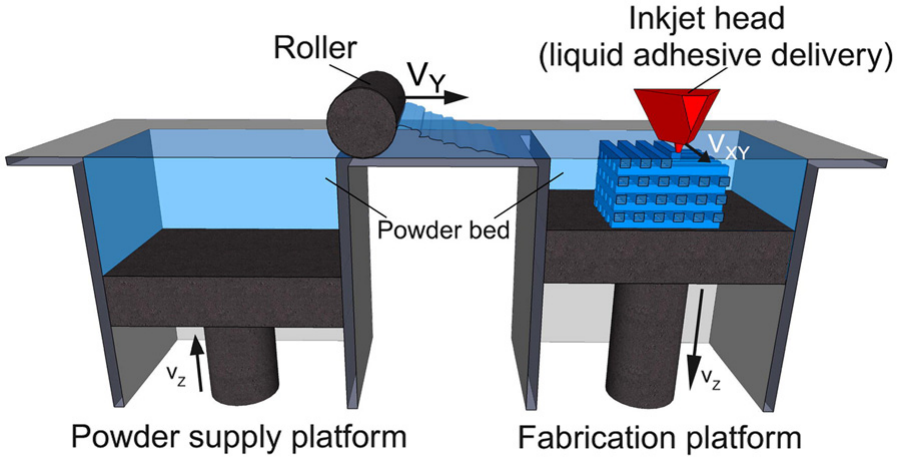
**3D Printing schematic.** 3D printing is a layer-by-layer process of depositing liquid binder onto thin layers of powder to create a 3D object. Reproduced with permission from [11].

Invented at the Massachusetts Institute of Technology, Three Dimensional Printing (3DP) fabricates 3D structures by inkjet printing liquid binder solution onto a powder bed [8-10]. A wide range of materials has been utilized in printing since most biomaterials exist in either a solid or liquid state. The process begins by spreading a layer of fine powder material evenly across the piston. The X-Y positioning system and the printhead are synchronized to print the desired 2D pattern by selective deposition of binder droplets onto the powder layer (Figure 1) [11]. The piston, powder bed, and part are lowered, and the next layer of powder is spread. The drop-spread-print cycle is repeated until the entire part is completed. Removal of the unbound powder reveals the fabricated part. The local composition can be manipulated by specifying the appropriate printhead to deposit the predetermined volume of the appropriate binder. The local microstructure can be controlled by altering the printing parameters during fabrication [12]. The incorporation of micro-channels effectively distributed additional seeding surfaces throughout the interior of the device, increasing the effective seeding density and uniformity. Patterned surface chemistry potentially offers spatial control over cell distribution of multiple cell type. This technology is limited by the competing needs between printhead reliability and feature resolution, as small nozzles can make finer features but are more prone to clogging. Current limitation in resolution is 100 μm for one-dimensional features (e.g. width of the thinnest printable line), and 300 μm for three dimensional features (e.g. thickness of thinnest printable vertical walls).

Fabrication of complex scaffolds such as internal channels or hanging features is easily achievable with this technique, since objects are being supported by surrounding unbounded powders. Kim et al. created highly porous scaffolds in combination with particulate leaching techniques by 3DP and demonstrated cell ingrowth into the scaffolds [13]. Also, room temperature processing conditions allow the incorporation of temperature sensitive materials such as pharmaceutical and biological agents into scaffolds [10]. Lam et al. fabricated starch-based scaffolds by printing distilled water, demonstrating the feasibility of using biological agents and living cells during fabrication [14]. Another favorable characteristic of this technology for tissue engineering is multi-“color” printing where each color ink can be positioned on a precise location. This feature offers the exciting potential to simultaneously arrange multiple types of cells, deposit multiple extra cellular matrix materials, and exert point-to-point control over bioactive agents for biological tissue manufacturing. In this respect, 3DP may be more flexible for printable material selection than other SFF technologies. A wide range of biological agents such as peptides, proteins (e.g. fibrinogen, collagen), polysaccharides (e.g. hyaluronan, alginate), DNA plasmids, and living cells have been printed with 3DP. Deposition of these biological materials requires modification of industrial 3DP machines. Cells in particular must be kept in a proper environment with appropriate temperature, oxygenation, and nutrient supply.

**Figure 2:**
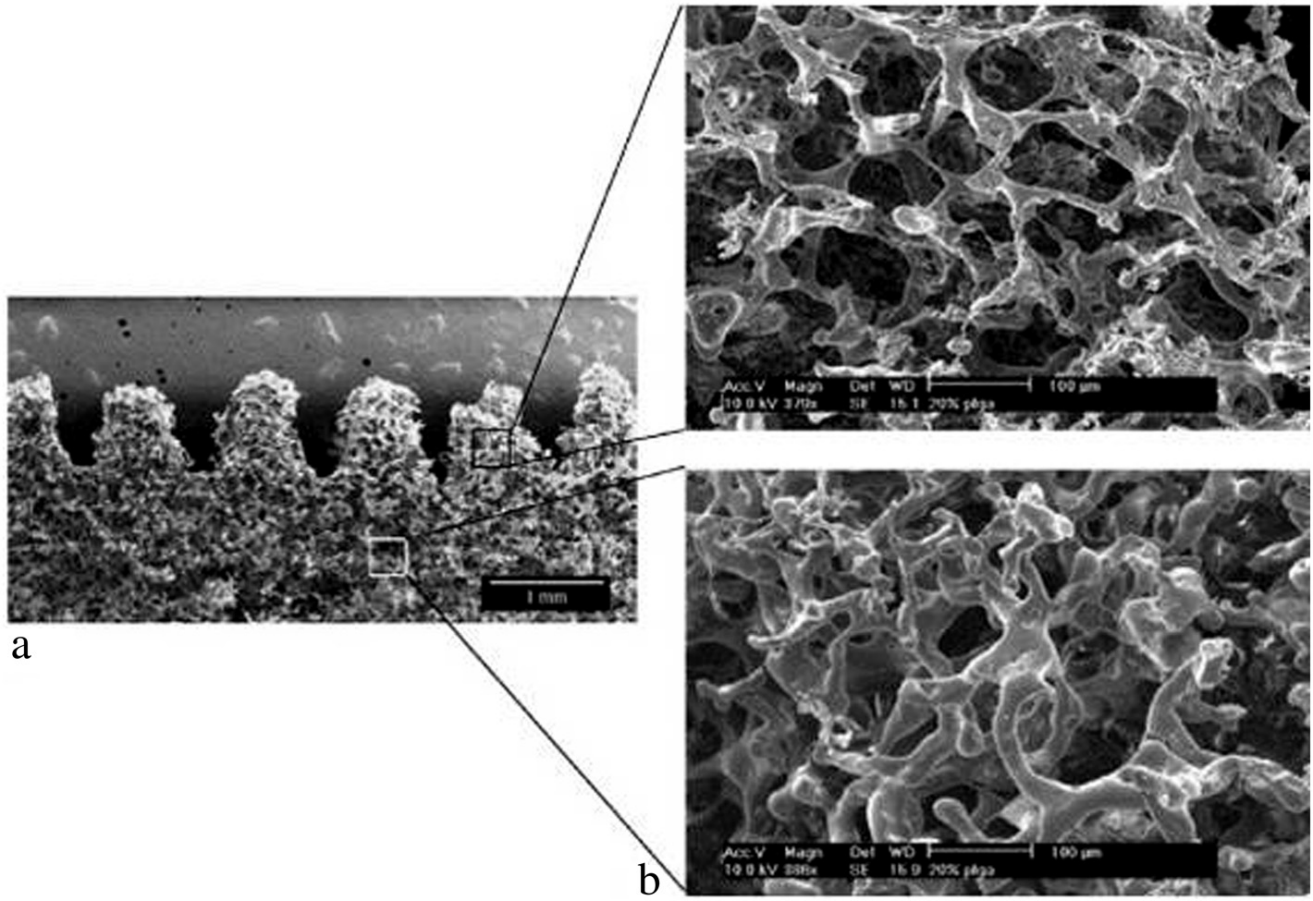
**PLGA scaffold with villi-shaped pillars created from indirect 3D Printing.** Scaffolds are created by packing a 3D printed mold with porogen and polymer dissolved in solvent by indirect 3DP. The resulting scaffolds have the desired villi-shaped pillars **(a)** and high porosity and interconnectivity **(b)**. Reproduced with permission from [17].

Other materials previously used in direct 3DP include powder composed of a synthetic polymer (i.e. poly (ε-caprolactone), polylactide–coglycolide or poly (L-lactic acid)) with organic solvent as binder [10,13,15] and natural polymer powder (i.e.starch, dextran and gelatin) with water as binder [14,16]. Indirect 3DP prints a mold which is then cast with the final polymer and porogen materials. Materials previously used in indirect 3DP to print the mold include commercially available plaster powder (i.e. calcium sulfate hemihydrate plaster powder) and water-based binder. The mold is then cast with a slurry of biodegradable polymer dissolved in solvent mixed with porogen (i.e.polylactide–coglycolide in chloroform mixed with NaCl) [17,18]. The resulting porous scaffold can be seen in Figure 2 with villi-shaped pillars [17]. Tissue engineers have used 3DP to fabricate porous ceramic scaffolds with fully interconnected channels directly from hydroxyapatite (HA) powder for bone replacement [16]. Customized anatomically shaped HA constructs can be fabricated based on medical information from a patient. This technology also allows a construction of a biphasic scaffold to regenerate hybrid tissue systems such as temporomandibular joint (TMJ). Sherwood et al. have developed osteochondral composite constructs in which the upper region is composed of D,L-PLGA/L-PLA with 90% porosity for cartilage regeneration, and the lower region is composed of a L-PLGA/TCP composite to maximize bone ingrowth [19]. A highly porous scaffold was created using this 3DP technology in combination with a particulate leaching technique.

**Figure 3:**
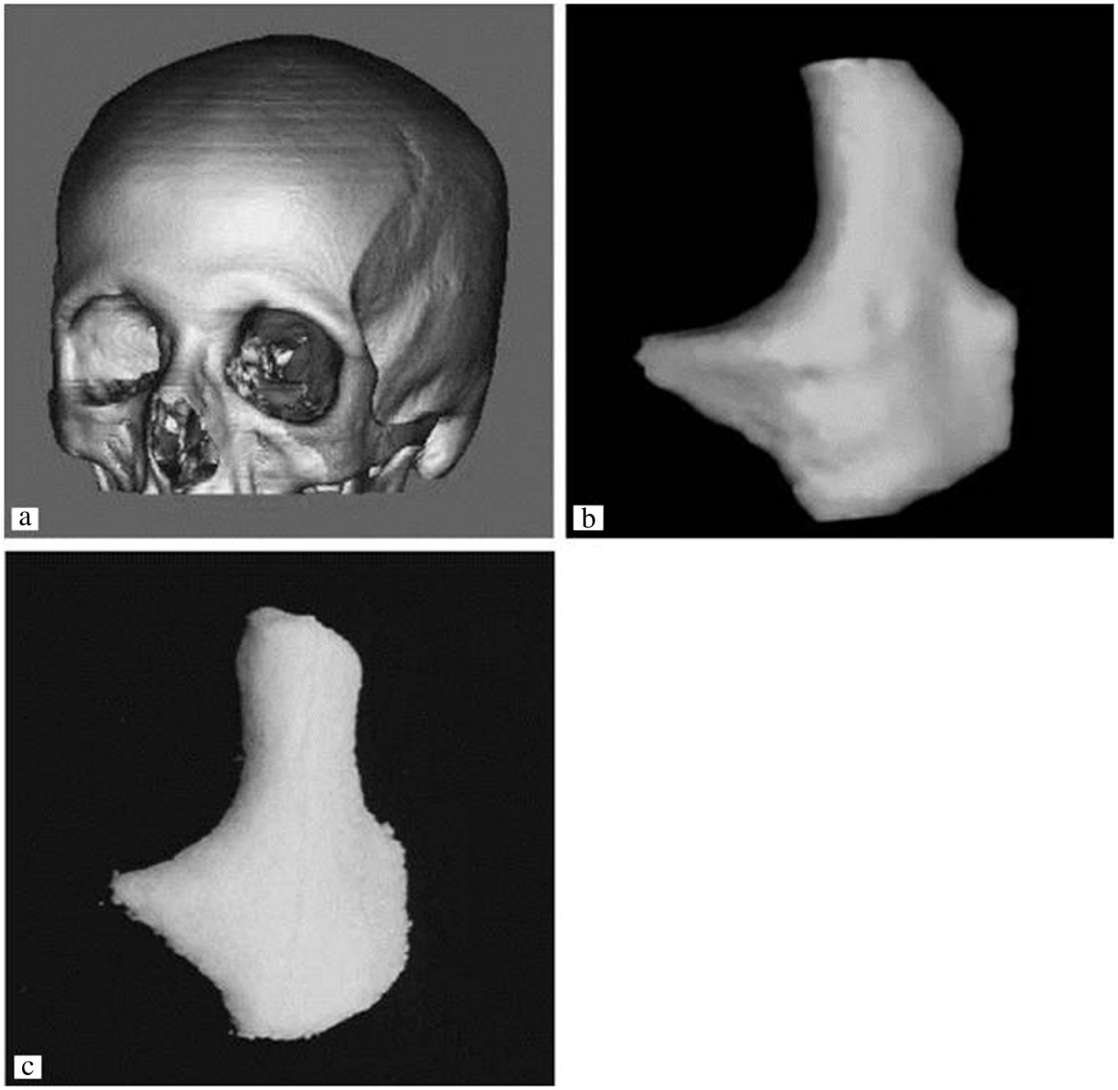
**3D printed scaffolds can be patient-specific.** A zygoma was generated from CT 2D images **(a,b)** and zygoma-shaped scaffold was produced from indirect 3DP **(c)**. Reproduced with permission from [17].

This problem was addressed by a practical, indirect 3DP protocol, where molds are printed and the final materials are cast into the mold cavity [17,18]. In the indirect technique, molds are printed using commercially available plaster powder, and biodegradable polymers are cast into the printed mold. Many different materials can be cast under the similar printing process parameters, whereas individual process parameters need to be optimized to maximize the build resolution in a conventional direct 3DP approach. This technology could be applied to treat patients with zygomatic bone fractures. Lee et al. demonstrated the ability of the indirect 3DP approach to build zygoma scaffold directly from CT data which can be seen in Figure 3 [17].

An advantage of direct 3DP is direct control over both the microarchitecture (i.e. pore size) and macroarchitecture (i.e. overall shape). Prints which use porogen as the powder result in high pore interconnectivity, uniform porosity, and defined pore size after leaching. This method has shown to fabricate scaffolds which can support hepatocyte ingrowth [13]. Unlike indirect 3DP, there are no limitations on the macroarchitecture and no need for demolding. One limitation of direct 3DP is that organic solvents can dissolve polymers used in most printheads. To overcome this limitation, investigators used stencils to pattern polymer solutions onto porogen particles (NaCl) to fabricate scaffolds [13]. However, the use of stencils prevents fabrication of highly complex shapes or small features. Organic solvent-compatible, high precision printheads are available but they are optimized for a narrow range of polymeric solutions. Another limitation of direct 3DP is that layer thickness must be greater than porogen particle size, and less than 150 μm maximum threshold to maintain interlayer connectivity and part strength during printing [12]. To overcome this porogen size limitation, larger pores must be printed. One drawback of 3DP is a limited available pore size in the final constructs when porogens are incorporated into powders prior to fabrication [15]. The shape complexity of scaffolds is also limited when the powder material is degradable polymer. Also, this 3DP approach for degradable polymer demands the use of organic solvents as liquid binders. Since organic solvents can dissolve most commercially available drop-on-demand printhead components, the reported studies required the use of custom machines, high resolution jets through stencils [15]. However, this approach is impractical for complicated structures. Indirect 3DP overcomes many of the limitations of direct 3DP. In the indirect technique, molds are printed using commercially available modeling materials such as plaster, and biodegradable polymers are cast into the printed mold. Many different materials can be cast under the similar printing process parameters, whereas individual process parameters need to be optimized to maximize the build resolution in a conventional direct 3DP approach. This technology could be applied to treat patients with zygomatic bone fractures. The use of aqueous binder allows the use of consumer grade inkjet printheads, and eliminates the need for stencils [17]. The porogen size is not limited since it is introduced into the mold cavity after printing, and does not affect printing resolution or layer interconnectivity. High materials flexibility with polymer-porogen combinations is possible due to independence from powder material properties. This method can be used to create small, high aspect ratio features (i.e. small intestine villi) or large scale, highly porous scaffolds (i.e. anatomically shaped zygoma scaffolds with pore sizes 300-500 μm) [18]. The limitations of indirect 3DP are 1) challenges in uniform, high density packing of porogen in complex features (i.e. intricate internal undercuts or intersecting channels) and 2) restrictions on shape or feature design due to difficulty demolding. Incomplete packing will result in loss of uniform microarchitecture and desired macroarchitecture.

The key advantages of 3DP are the wide range of materials able to be used due to room temperature processing and the material used in powder form, ability to print overhangs and internal architecture, and microstructure control. The disadvantages of 3D Printing are the limited use of organic solvents as binders due to dissolving of commercial printheads and difficulty in removing unbound powder from small or curved channels.

#### Recent material and technology advances

3DP materials include calcium polyphosphate and PVA [20], HA and TCP [21-25], TCP [26-29], TCP with SrO and MgO doping [30,31], HA and apatite–wollastonite glass ceramic with water-based binder [32], calcium phosphate with collagen in binder [33], PLGA [34], and Farringtonite powder (Mg_3_(PO_4_)_2_) [35]. Materials used in indirect 3DP gelatin preforms replaced with PCL and chitosan [36]. In vitro studies with bovine chondrocytes for articular cartilage tissue engineering [20], bone tissue engineering [21,22,25,26,37], monocytic cells from the RAW 264.7 cell line [22], human osteoblasts [23,29,32,34], C2C12 pre-myoblastic cell line [24], and bone marrow stromal cells [36]. In vivo studies have been performed with rabbit calvarial bone [26], rabbit tibia bone and porcine maxillary bone [24], rat femoral defects [28,30], mouse femoral defects [33], and rabbit femoral bone [31].

### Fused deposition modeling

#### Technology description and application

**Figure 4:**
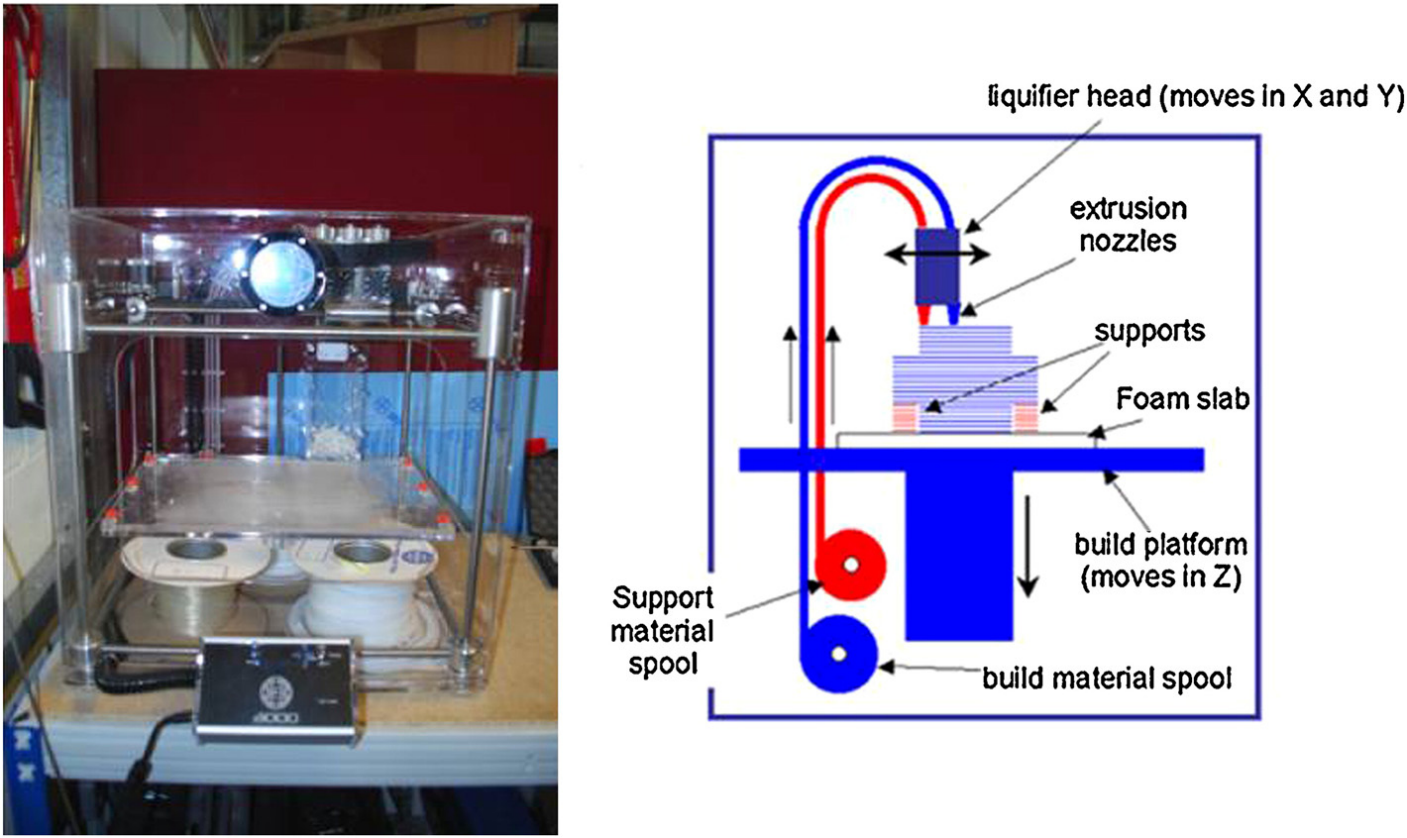
**Fused deposition modeling schematic.** In fused deposition modeling, a filament of thermoplastic is heated into liquid and extruded through a nozzle in a specific lay-down pattern to create a scaffold. Reprinted with permission from [39].

Fused deposition modeling (FDM) is the deposition of molten thermoplastic materials through two heated extrusion heads with a small orifice in a specific laydown pattern [38]. One nozzle deposits the thermoplastic material and the second deposits temporary material to support cantilevers. In FDM, one of the traditional methods melts thermoplastic polymer into a semi-liquid state and the head extrudes the material onto the build platform (Figure 4) [39]. The part is built in a layer-by-layer fashion where the layers are fused together. Since multiple extrusion nozzles could be used in FDM, each with a different material, there is no theoretical restriction on compositional gradients in all three dimensions for FDM. However, this has not been reduced to practice.

The most important material selection criteria for FDM materials are heat transfer characteristics and rheology (behavior of liquid flow). Thermoplastics are commonly used due to the low melting temperature. PVC, nylon, ABS, and investment casting wax have been successfully used. For bioapplications, PCL is commonly used due to its low melting temperature of ~60°C, low glass transition temperature of -60°C, and high thermal stability [38,40]. PLGA previously has been used with FDM to create scaffolds, however, the high glass transition temperature of PLGA (40-60°C) makes processing PLGA challenging with a higher extrusion temperature required [41,42]. The material is heated to ~110-140°C to create the right material flow properties for extrusion from the nozzle and fusion of the layers [38,40-42]. Rheological modifiers can be used but must be biocompatible.

Controllable variables are raster thickness, raster gap width (space between rasters), raster angle, and layer thickness (dependent on extrusion tip diameter). This results in scaffolds with controlled pore size, morphology, and interconnectivity. The extruded molten liquid must be hot enough to rapidly induce fusion with previously extruded material and solidify quickly to minimize flow and feature size. In addition, the viscosity of the material is critical to be both high enough to allow extrusion through a fine nozzle and low enough to

Scaffolds with biocompatible materials have been made with different pore morphology and channel sizes by controlling the x-y movement of the extrusion head [38]. Materials can also be combined in this technology such as poly(ethylene glycol) terephthalate/poly(butylene terephthalate) or polypropylene/TCP [43,44]. Other composites such as PCL/HA or PCL/TCP are used with FDM due to favorable mechanical and biochemical properties for bone regeneration [45].

The key advantages of FDM are high porosity due to the laydown pattern and good mechanical strength. A challenge for FDM is the limitation to thermoplastic materials with good melt viscosity properties which have high enough viscosity to build but low enough viscosity for extrusion. Also, these properties have limited shape complexity for biological scaffolding materials and typically result in relatively regular structures [40]. It should be noted that geometric complexity is not limited for FDM using industrial materials which are selected to have optimal thermal and rheological properties but lack biocompatibility. Another disadvantage for FDM is the inability to incorporate living cells or temperature sensitive biological agents during extrusion due to the high processing temperature.

#### Recent material and technology advances

FDM has commonly used biocompatible polymers with low melting temperatures. Materials used in FDM to create scaffolds are PCL and bioactive glass composites [46], L-lactide/e-caprolactone [46], PLGA with collagen infiltration [47], PCL-TCP with gentamicin [48], PCL-TCP [49], PLGA-TCP and coated with HA[42] , PCL-PLGA-TCP [50], PLGA-PCL [51], PCL coated with gelatin [52], PCL [53,54], PMMA [55], and PLA [46]. In vitro studies have been performed with porcine chondrocytes [47], mouse pre-osteoblasts [52], and bone marrow-derived mesenchymal stem cell [53]. In vivo studies with murine animal models for wound healing [48], human patient for craniofacial defect [49], and rabbit bone defect [42,50]. Applications include cartilage tissue engineering, antibiotic delivery system[48], osseous craniofacial defects in humans [49,55], and bone tissue engineering [13].

While the number of FDM filaments are increasing every month, the material choices pale in comparison to the total number of theromoplastics that can be formed by conventional injection molding. One recent advance may vastly increase the range of materials available for 3D Printing, and transform it from a prototyping method to a viable manufacturing method is to incorporate precision injection molding into 3D Printing gantry. This combination has significant potential because it can process most thermoplastics that exist as conventional injection molding pellets, without pre-processing into fine powders or traditional FDM filaments. It is essentially mould-free injection molding of final structures, making it feasible to fabricate one-of-a-kind, one patient at a time medical device [56].

### Stereolithography

#### Technology description and application

**Figure 5:**
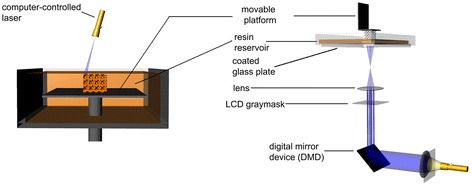
**Stereolithography schematic.** Stereolithography is the polymerization of photocurable resin by a bottom-up system with scanning laser (left) or top-down setup with digital light projection (right). Reproduced with permission [59].

Stereolithography (SLA) is the regarded as the first rapid prototyping process and was developed in the late 1980s [57]. The original SLA rasters a HeCd-laser beam to spatially control the polymerization of photocurable resin in 2D patterns [58]. After each layer is cured, the platform with the cured structure attached then lowers in the bottom-up approach and another layer of uncured liquid resin spreads over the top. The topmost layer is now ready to be patterned. For the top-down approach, light is projected onto a transparent plate initially positioned near the bottom of the vessel holding the liquid resin (Figure 5) [59]. After a layer is patterned through the transparent plate, the cured structure is detached from the transparent plate. The cured structure is raised to allow uncured liquid resin to fill the space between the structure and transparent plate. The next layer is now ready to be patterned. Since rastering a laser beam can be slow, especially for large parts, the masked lamp technique was developed to cure an entire layer of photopolymers at a time. After the structure is built, the unpolymerized liquid resin is removed by draining. Post-curing in a UV oven converts any unreacted groups and strengthens the part [60].

Kinetics of the curing reactions occurring during polymerization is critical. This affects the curing time and the thickness of the layer polymerized. The kinetics can be controlled by the power of the light source, the scanning speed and the chemistry and amount of the monomer and photointiators. In addition, UV absorbers can be added to the resin to control the depth of polymerization [61].

Materials must have photocurable moieties for photocrosslinking. Typical materials used in STL include acrylics and epoxies. For tissue engineering applications, there are very few biodegradable and biocompatible biomaterials that are dimensionally stable during photopolymerization. Photocrosslinkable poly(propylene fumarate) (PPF) [62] is commonly used in SLA and has been used to fabricate complex 3D scaffolds with controlled microstructures for reconstruction of rabbit cranial defects [58]. PPF requires a reactive diluent, such as diethyl fumarate or N-vinyl-2-pyrrolidone, to reduce the viscosity of the resin for proper processing conditions [63]. These diluents introduce significant amounts of a non-degradable component. Resins with and without bioceramic dispersions have been processed by SLA.

Medical applications of SLA include the fabrication of anatomical models for pre-surgical planning, and indirect fabrication of medical devices by using the SLA patterns for molds (e.g. filling a SLA structure to use as a negative mold) [64,65] . Titanium dental implant components have been fabricated by electrical discharge machining of titanium ingot based on a SLA model.

The advantages of SLA are the ability to create complex shapes with internal architecture, ease of removal of unpolymerized resin, and extremely high feature resolution (~1.2 um) [66]. The main disadvantage of SLA is the scarcity of biocompatible resins with proper SLA processing properties. Additional challenges are the use of photointiators and radicals which may be cytotoxic (with long processing times), entrapment of unreacted monomer and residual photoinitiator, and inability to create compositional gradients along horizontal planes. Photopolymerized resin also has poor mechanical properties that are needed for hard tissue engineering. Lastly, temporary support structures must be incorporated into the CAD model to fabricate unsupported features (e.g. overhangs, cantilevers). Complete removal of support structures may be difficult.

#### Recent material and technology advances

Recent improvements for SLA have been increasing the library of photocrosslinkable polymers and the use of multiple resins for one build. Over the last few years, more polymers are synthesized containing aliphatic polyesters which allow for biodegradation. The resulting macromer is then acrylated to allow for photocrosslinking capability. The use of multiple resins for one build was shown with patterning PEG-DMA and PEG-DA with fluorescently labeled dextran, fluorescently labeled bioactive PEG or bioactive PEG in different regions of the scaffold [67]. When changing material, the scaffold would be removed from the pool of resin, rinsed with distilled water, and new resin added to the vat. A fixture was used to maintain X-Y registration of the scaffold to ensure alignment of layers. Dynamic mask projection SLA has been able to achieve a lateral resolution of ∼ 2 μm, and vertical resolution of ∼ 1 μm for PPF resin [68]. The microstructures able to be produced with this technology are extremely detailed although there are still challenges of creating horizontal channels and preventing shrinkage of structures.

SLA recently has increased the library of resins with biodegradable moieties and the encapsulation of cells during processing. Novel macromers synthesized include segments of PCL (three-armed hydroxyl-terminated) [69] or poly(d,l-lactide) [63,70,71]. Photo-curable poly(D,L-lactide) (PLLA) resin without the use of reactive diluents has been developed and applied in SLA [70]. The end groups are modified to acrylate or methacrylate to allow for photo-crosslinking capability. Another resin recently used in making SLA scaffolds is PPF-DEF [72,73] and PPF-DEF with BMP-2 loaded PLGA microspheres [74]. PPF-DEF or PPF-DEF with HA is used in μSL (<5 μm resolution) although shrinkage of the polymer occurs to cause warping of the parts [68,75]. Poly(trimethylene carbonate) macromers have been used for flexible, elastic applications with stiffness of 22-156 kPa [76] such as for cartilage tissue engineering [77]. Stiffer structures have been studied in vitro with mouse pre-osteoblasts [70], human umbilical vein endothelial cells [78], rat bone marrow cells [73], MC3T3-E1 pre-osteoblasts [63,72,74], and human mesenchymal stem cells[71]. A large application of SLA is bone tissue engineering [79] and in vivo studies have shown promoted bone formation in rat cranial defects [74]. For softer, flexible applications such as cartilage tissue engineering in vitro studies have been performed with bovine chondrocytes [77]. Cell seeding and culturing was found to be improved with scaffolds with SLA-controlled pore network architecture compared to scaffolds made from salt leaching with poly(d,l-lactide) oligomers and PFF-DEF [73,80]. Cell encapsulation during SLA has been shown with PEG-DA with NIH/3T3 cells [81] and PEG-DMA with human chondrocytes although with an inkjet printer [82].

### Selective laser sintering/melting

#### Technology description and application

**Figure 6:**
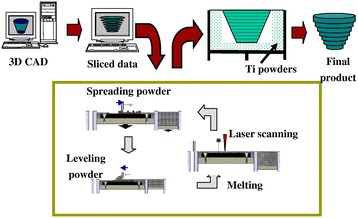
**Selective laser sintering schematic.** Selective laser sintering uses a laser to fuse together powder particles to create a 3D scaffold. Thin layers of powder are spread between each fused layer. Reprinted with permission from [83].

Selective laser sintering (SLS) was develop by the University of Texas in 1989. SLS is similar to 3DP in binding together powder particles in thin layers except a CO_2_ laser beam is used (Figure 6) [83]. The laser scans the surface of the powdered polymer particles in a specific 2D pattern to sinter by heating them above the glass transition temperature. During sintering, molecular diffusion along the outermost surface of the particle lead to neck formation between neighboring particles. After one layer is created, the piston containing the part is lowered and a fresh layer of powder material is rolled across the top surface. The subsequent layer is formed and is bound to the previous layer. Unbound, loose powder is removed after the part is completed and is heat treated to achieve full density. Temporary support structures are not needed, unlike in SLA, since unbound solid particles support any cantilever structures. Since sintering does not result in complete melting of the powder particle, the porosity between the original particles can be preserved, and a wide range of pure and mixture of materials can be processed.

While solid state sintering can be achieved for most materials between 0.5-1 T_melting_, selective laser melting (SLM) and electron beam melting (EBM) use intense energy to heat the powder above T_melting_ to completely fuse the particles into one fully-dense, consolidated structure. In In practice, melting is more easily accomplished if all powder has a single melting point, and is therefore more easily accomplished with pure metals than with alloys due to variation in liquid metal flow behavior, surface tension, and laser-material interactions. Therefore, the range of materials for SLM is more limited than SLS.

The resolution of features is determined by powder particle size, focused laser beam diameter and heat transfer in the powder bed. The limit to particle size is 10 um due to poor spreading and sintering too quickly causing edge inaccuracies. Materials commonly used are PCL and a combination of polyether ether ketone and hydroxyapatite [84-87]. With biomaterials, thin solid disks are commonly made but feature are made on the ~400-500 μm scale.

Previously coated ceramic powders and thermoplastics have been used in SLS. Intermediate binding materials are required because of an excessively high glass transition temperature and the melting point of ceramic powder. The intermediate binding materials would melt before the ceramic powder and fuse together the ceramic particles. Tan et al. fabricated calcium phosphate bone implants by sintering calcium phosphate powder coated with polymer [88]. After the part is built and excess powder removed, post processing (e.g. extra sintering in an oven) increases part strengths but can cause shrinkage of the parts. A biocomposite blend of polyvinyl alcohol (PVA) and hydroxyapatite (HA) was also used in SLS [89]. HA particles were coated with a water-soluble PVA via spray-drying or physical blending. These parts were used for craniofacial and joint defect applications. Williams et al. fabricated PCL scaffolds with porous architecture and sufficient mechanical properties for bone tissue engineering applications [90].

This technique is also feasible with medical data to create anatomy specific structures. A mandibular condyle scaffold was demonstrated in this technique using CT data from a pig condyle [90]. The integration of computational design and SLS techniques enables the ability to fabricate scaffolds that have anatomically shaped external architectures and porous interior structure. FDA clearance was recently awarded for the use of SLS to process medical grade polyether ether ketone (PEEK) to make custom craniofacial implants. More recently, SLM was used to create the first patient-specific, ready for implantation titanium mandible that accepts dental implants to support a mandibular denture [91].

They key advantage of SLS/SLM/EBM is the ability to directly make metallic implants that promote either bone ingrowth and regeneration for load-bearing applications in which high fracture toughness and mechanical strength are needed. Even for non-load bearing applications, polymers can be processed without the use of organic solvent. It is slightly easier to achieve compositional gradients in SLS than SLA by spreading different powder between different vertical layers, but compositional gradients in the horizontal plane is very limited. The main disadvantages are limited materials which fuse but do not decompose under the laser beam (high temperatures) and the post processing needed to remove trapped powder. Another limitation is the condution and diffusion of laser heat causes unwanted fusion of neighboring powder particles, limiting the resolution of final features. Lastly, smaller pore sizes are limited since the created pores depend on the particle size of the powder used. Powder particles too small cannot be used due to poor spreading from powder clumping.

#### Recent material and technology advances

Recent advances of SLS have been the ability to produce lower stiffness scaffolds and higher resolution features. PCL scaffolds have been produced at lower stiffness of 300-400 kPa [87] than reported before at 14.9-113.4 MPa [85,86,90,92]. This lower stiffness allows for applications of soft tissue engineering such as cardiac tissue.

Work has been done to streamline the CAD/CAM process of making functionally graded scaffolds (FGS, changing stiffness within a part) by using a library of polyhedrals to control the porosity. The porosity then processed is related to the stiffness of the scaffold and demonstrated with PCL in SLS [93]. A thorough review on the development of the design of microarchitecture can be found [94]. In addition, FEA has been used to help design microarchitecture and predict mechanical properties for SLS [92,95].

For SLS, common materials used are PCL and HA [92,96,97], PCL and β-TCP with collagen coating [98], Ca-P/PHBV and CHAp/PLLA [99,100], and PVA [101]. To demonstrate encapsulation of biomolecules, BSA was encapsulated in Ca-P/PHBV microparticles and processed [102]. In vitro studies have been performed with C2C12 myoblast cells for cardiac tissue engineering [87], SaOS-2 cells [99], human bone marrow stromal cells [103], and human osteoprogenitor cells [52], porcine adipose-derived stem cells [98,104], and MG-63 [101] for bone tissue engineering. In vivo studies have been performed in nude mice showed better woven bone and vascular tissue formation [98]. Applications are bone tissue engineering and interbody cages for spinal fusions [97].

### 3D Plotting/Direct-write bioprinting

#### Technology description and application

3D plotting was developed at the Freiburg Materials Research Center in 2000 to create soft tissue scaffolds. 3D plotting is based on extruding a viscous liquid material (generally a solution, paste, or dispersion) from a pressurized syringe into a liquid medium with matching density. The material is deposited in one long continuous strand or in individual dots from a nozzle or syringe to create a desired 3D shape of ceramics, polymers, or hydrogels [105]. The process can be at room temperature or at elevated temperatures, but does not involve thermoplastics as in FDM.

This SFF method is particularly applicable for natural biomaterials to create hydrogels. Landers et al. used thermoreversible natural polymers such as agar and gelatin in solution. The solution is heated and extruded at ~80°C into a cooler liquid medium (~20°C) of gelatin or silicone oil to quickly solidify the heated solution. [106,107]. Another approach is to extrude polymers into a liquid medium containing reactants for crosslinking. An example material is extruding gelatin into a Ca^2+^ reservoir for microvasculature [108]. For other materials such as TCP, a solution is made with water, extruded from a syringe, and then lyophilized to remove the liquid [109]. The resulting diameter of each strut was ~400 μm.

The key advantages are material flexibility and room temperature processing (if applicable). In addition, many of the other SFF technologies cannot use natural polymers due to processing conditions. One key disadvantage is the difficulty in fabricating complex shapes with overhangs since a temporary, sacrificial material is needed. In addition, hydrogels created in this method have low stiffness which may result in collapse of structures or limitations on complexity of shapes.

**Figure 7:**
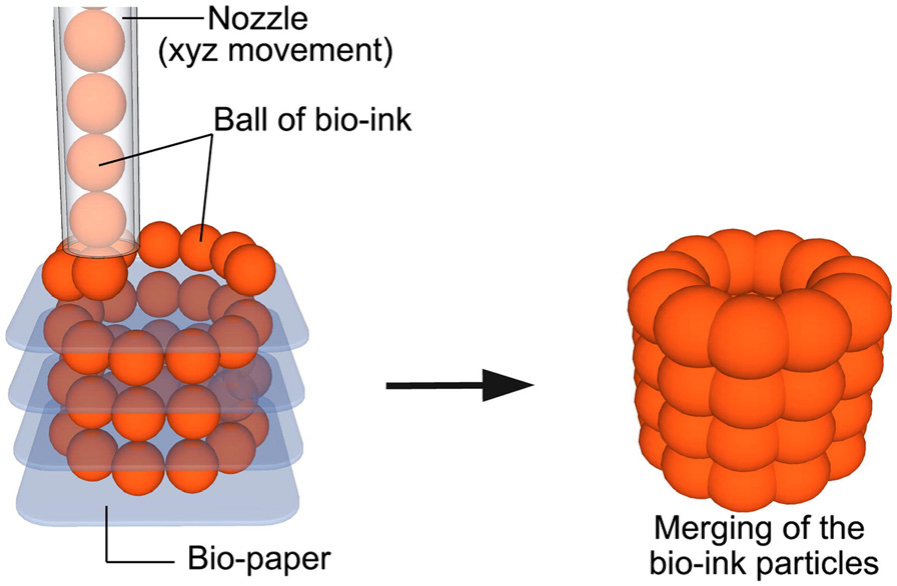
**Bioprinting schematic.** In bioprinting, small balls of bioink composed of cells and hydrogel materials (e.g. alginate or decellularized extracellular matrix) are printed in a desired shape. Reproduced with permission from [11].

Similarly, bioprinting is the fabrication of hydrogel structures with direct incorporation of cells (Figure 7). Cells are added during processing in cell printing strategies such as alginate-cell (bovine chondrocytes) solution extruded from a syringe [110], electrostatically driven inkjet printing of bovine vascular endothelial cells in culture medium [111], laser-guided direct writing of embryonic chick spinal cord cells [112], and laser-induced forward transfer of cells suspended in alginate [113]. This technology provides a controlled spatial distribution of cell or growth factors as well as the scaffold structures. However, this fabrication technique is generally limited to hydrogel materials such as alginate and fibrin, which may not be ideal for the implantation in biological environments that require strong mechanical properties. Example applications are rat smooth muscle cell-laden collagen droplets (650 μm diameter) to create specific cell spatial patterns in 3D [114]. This SFF method is especially good for low viscosity materials and the buoyancy due to the density matching of the extruded material to the liquid medium prevents collapse of the shape. The strand thickness can be varied by material viscosity, deposition speed, extrusion tip diameter, and applied pressure.

The key advantages of bioprinting are the room temperature processing (if applicable), direct incorporation of cells, and homogenous distribution of cells. The key disadvantages are limited mechanical stiffness, critical timing of gelation time, specific matching of material and liquid medium densities to preserve shapes, and low resolution. Further development of materials that are optimal for biofactor printing, and next generation printheads that can separately deposit multiple biofactors and materials onto the same platform, will provide the potential to create constructs satisfying complex biological requirements of tissue engineering scaffolds.

#### Recent material and technology advances

Bioplotting materials include PLGA, TCP, collagen and chitosan [109], chitosan [115], collagen-alginate-silica composites coated with HA [116], soy protein [117,118], and agarose with gelatin [107]. In vitro studies have been performed with mouse pre-osteoblasts [116] and human mesenchymal stem cells [117]. In vivo studies have been performed in ovine cavalarial defects [109]. Applications include bone tissue engineering [109,116], tissue regeneration [118].

Bioprinting materials are agarose with human umbilical vein smooth muscle cells (HUVSMCs) and human skin fibroblasts (rods) [119], gelatin-HA-tetraPEG-DA with NIH 3T3s (rods) [120], rat primary bladder smooth muscle cells in collagen droplets [114], human microvascular endothelial cells in fibrin (inkjet printer) [121], and alginate droplets [108]. Applications are mainly for vascular tissue engineering [108,119-121].

Recent studies show the ability to bioprint single cells and cell-laden hydrogel-PCL scaffolds. High throughput printing of single-cell arrays has been shown with “Block-Cell-Printing” [122]. Microfluidic arrays of hook-shaped traps are used to trap single cells. Trapped cells can be paired and separated 5 μm to study cell communication. In this study, trapped primary rat cortical neurons were cultured and cells exhibited neuronal morphology. Ahn et al. bioprinted high density cell-laden hydrogels by extruding a 4°C cell-alginate solution onto a -10°C stage to create a structure [123]. The alginate was crosslinked to provide strength by incubating the structure in a CaCl_2_ solution. Good cell viability of was shown for human mesenchymal stem cells and human osteoblast-like cells after processing. Lastly, a layer-by-layer process has alternately deposited chondrocyte-laden hydrogel droplets (alginate or decellularized extracellular matrix bioink) and PCL in a layer-by-layer process to create a 3D structure [124-126] (Figure 7).

Recent advances in biofactor printing technology allow the simultaneous printing of pharmaceutical and biological agents during fabrication. Xu et al. demonstrated that inkjet printing technology can precisely place the cells and proteins into 3D alginate structures [127].

## Future direction

Additional progress for 3D Printing technologies is needed for increasing resolution without sacrificing shape, strength, and handability of scaffolds. Anatomical features and tissue architecture may have details on the scale of hundreds of microns (e.g. villi of the small intestine with ~500 um diameters). Diffusion consumption modeling has shown a 200 μm limit in scaffolds for oxygen transport to cells, resulting in a maximum of 400 μm diameter features for cell survival [128]. For both SLS and 3DP, there is a challenge with creating stronger structures without increasing dimensions. To create small features which survives the fabrication process, powder particles much be bound together tightly. By increasing the strength of the laser for SLS or amount of binder for 3DP, additional powder particles would bind and therefore increase the dimensions. Additional work is needed to move SLS and 3DP to resolutions below 400-500 μm. In addition, unbound trapped powder is difficult to remove from small channels. Future work is needed to create powder that is easily removable with traditional methods of high-pressured air. One strategy is to create spherical powder particles which would facilitate removal in tight spaces.

While SLA can reach extremely high resolutions, there are a limited number of biodegradable, biocompatible resins. Advances have been made to synthesize new macromers with biodegradable moieties, however, these materials have not been FDA approved. FDM, SLS, and 3DP are able to use polymers such as PLGA, PLLA, and PCL without chemical modification which will help expedite future FDA approval for biomedical devices.

Although macro and microarchitecture has made great strides in the past five years, additional work should focus on the nanoarchitecture (e.g. biochemical molecules). Due to harsh processing conditions of SFF methods (e.g. heat, organic solvent), biochemical molecules are not generally incorporated directly into the scaffold. While biochemical molecules can be coated onto structures in post-processing, there is a need for sustained growth factor release over time. Therefore, strategies to incorporate biochemical molecules directly into scaffolds for prolonged release will be needed.

Although the focus of this review article is on the fabrication techniques and biomaterials used in 3DP, the degradation kinetics and byproducts of the materials are in fact a very significant problem in 3D scaffolds due to mass transport limitations within thick scaffolds. This is a moving boundary diffusion-reaction problem that even without biodegradable biomaterials can result in hypoxia and acidosis within the scaffolds. The release of acidic degradation products is expected to worsen the acidosis which may harm the seeded cells and/or the surrounding cells”.

## References

[1] Karande TS, Ong JL, Agrawal CM (2004). Diffusion in musculoskeletal tissue engineering scaffolds: design issues related to porosity, permeability, architecture, and nutrient mixing. Ann Biomed Eng.

[2] Hollister SJ (2005). Porous scaffold design for tissue engineering. Nat Mater.

[3] Stevens MM, George JH (2005). Exploring and engineering the cell surface interface. Science.

[4] Winder J, Bibb R (2005). Medical rapid prototyping technologies: state of the art and current limitations for application in oral and maxillofacial surgery. J Oral Maxillofac Surg.

[5] Colin A, Boire J-Y (1997). A novel tool for rapid prototyping and development of simple 3D medical image processing applications on PCs. Comput Methods Programs Biomed.

[6] Winder J (1999). Medical rapid prototyping and 3D CT in the manufacture of custom made cranial titanium plates. J Med Eng Technol.

[7] Hollister S, Maddox R, Taboas J (2002). Optimal design and fabrication of scaffolds to mimic tissue properties and satisfy biological constraints. Biomaterials.

[8] Cima MJ, Sachs E, Cima LG, Yoo J, Khanuja S, Borland SW, et al. Computer-derived microstructures by 3D printing: bio-and structural materials. Solid Freeform Fabr Symp Proc: DTIC Document; 1994. p. 181-90

[9] Griffith LG, Wu B, Cima MJ, Powers MJ, Chaignaud B, Vacanti JP (1997). In Vitro Organogenesis of Liver Tissuea. Ann N Y Acad Sci.

[10] Wu BM, Borland SW, Giordano RA, Cima LG, Sachs EM, Cima MJ (1996). Solid free-form fabrication of drug delivery devices. J Control Release.

[11] Billiet T, Vandenhaute M, Schelfhout J, Van Vlierberghe S, Dubruel P (2012). A review of trends and limitations in hydrogel-rapid prototyping for tissue engineering. Biomaterials.

[12] Wu BM, Cima MJ (1999). Effects of solvent‐particle interaction kinetics on microstructure formation during three‐dimensional printing. Polymer Eng Sci.

[13] Kim SS, Utsunomiya H, Koski JA, Wu BM, Cima MJ, Sohn J (1998). Survival and function of hepatocytes on a novel three-dimensional synthetic biodegradable polymer scaffold with an intrinsic network of channels. Ann Surg.

[14] Lam CXF, Mo XM, Teoh SH, Hutmacher DW (2002). Scaffold development using 3D printing with a starch-based polymer. Mater Sci Eng C.

[15] Zeltinger J, Sherwood JK, Graham DA, Müeller R, Griffith LG (2001). Effect of pore size and void fraction on cellular adhesion, proliferation, and matrix deposition. Tissue Eng.

[16] Seitz H, Rieder W, Irsen S, Leukers B, Tille C (2005). Three‐dimensional printing of porous ceramic scaffolds for bone tissue engineering. J Biomed Mater Res B Appl Biomater.

[17] Lee M, Dunn JCY, Wu BM (2005). Scaffold fabrication by indirect three-dimensional printing. Biomaterials.

[18] Lee M, Wu BM, Dunn JCY (2008). Effect of scaffold architecture and pore size on smooth muscle cell growth. J Biomed Mater Res A.

[19] Sherwood JK, Riley SL, Palazzolo R, Brown SC, Monkhouse DC, Coates M (2002). A three-dimensional osteochondral composite scaffold for articular cartilage repair. Biomaterials.

[20] Shanjani Y, Croos D, Amritha J, Pilliar RM, Kandel RA, Toyserkani E (2010). Solid freeform fabrication and characterization of porous calcium polyphosphate structures for tissue engineering purposes. J Biomed Mater Res B Appl Biomater.

[21] Seitz H, Deisinger U, Leukers B, Detsch R, Ziegler G (2009). Different Calcium Phosphate Granules for 3‐D Printing of Bone Tissue Engineering Scaffolds. Adv Eng Mater.

[22] Detsch R, Schaefer S, Deisinger U, Ziegler G, Seitz H, Leukers B. In vitro-osteoclastic activity studies on surfaces of 3D printed calcium phosphate scaffolds. J Biomater Appl. 2010. 10.1177/0885328210373285 20659962

[23] Warnke PH, Seitz H, Warnke F, Becker ST, Sivananthan S, Sherry E (2010). Ceramic scaffolds produced by computer‐assisted 3D printing and sintering: Characterization and biocompatibility investigations. J Biomed Mater Res B Appl Biomater.

[24] Abarrategi A, Moreno-Vicente C, Martínez-Vázquez FJ, Civantos A, Ramos V, Sanz-Casado JV (2012). Biological properties of solid free form designed ceramic scaffolds with BMP-2: in vitro and in vivo evaluation. PLoS One.

[25] Becker ST, Bolte H, Krapf O, Seitz H, Douglas T, Sivananthan S (2009). Endocultivation: 3D printed customized porous scaffolds for heterotopic bone induction. Oral Oncol.

[26] Tamimi F, Torres J, Gbureck U, Lopez-Cabarcos E, Bassett DC, Alkhraisat MH (2009). Craniofacial vertical bone augmentation: a comparison between 3D printed monolithic monetite blocks and autologous onlay grafts in the rabbit. Biomaterials.

[27] Butscher A, Bohner M, Roth C, Ernstberger A, Heuberger R, Doebelin N (2012). Printability of calcium phosphate powders for three-dimensional printing of tissue engineering scaffolds. Acta Biomater.

[28] Tarafder S, Balla VK, Davies NM, Bandyopadhyay A, Bose S (2013). Microwave‐sintered 3D printed tricalcium phosphate scaffolds for bone tissue engineering. J Tissue Eng Regen Med.

[29] Santos CF, Silva AP, Lopes L, Pires I, Correia IJ (2012). Design and production of sintered β-tricalcium phosphate 3D scaffolds for bone tissue regeneration. Mater Sci Eng C.

[30] Tarafder S, Davies NM, Bandyopadhyay A, Bose S (2013). 3D printed tricalcium phosphate bone tissue engineering scaffolds: effect of SrO and MgO doping on in vivo osteogenesis in a rat distal femoral defect model. Biomater Sci.

[31] Tarafder S, Dernell WS, Bandyopadhyay A, Bose S. SrO‐and MgO‐doped microwave sintered 3D printed tricalcium phosphate scaffolds: Mechanical properties and in vivo osteogenesis in a rabbit model. J Biomed Mater Res Part B: Appl Biomat. 2014 10.1002/jbm.b.33239 25045131

[32] Suwanprateeb J, Sanngam R, Suvannapruk W, Panyathanmaporn T (2009). Mechanical and in vitro performance of apatite–wollastonite glass ceramic reinforced hydroxyapatite composite fabricated by 3D-printing. J Mater Sci Mater Med.

[33] Inzana JA, Olvera D, Fuller SM, Kelly JP, Graeve OA, Schwarz EM (2014). 3D printing of composite calcium phosphate and collagen scaffolds for bone regeneration. Biomaterials.

[34] Ge Z, Wang L, Heng BC, Tian X-F, Lu K, Fan VTW (2009). Proliferation and differentiation of human osteoblasts within 3D printed poly-lactic-co-glycolic acid scaffolds. J Biomater Appl.

[35] Klammert U, Vorndran E, Reuther T, Müller FA, Zorn K, Gbureck U (2010). Low temperature fabrication of magnesium phosphate cement scaffolds by 3D powder printing. J Mater Sci Mater Med.

[36] Lee J-Y, Choi B, Wu B, Lee M (2013). Customized biomimetic scaffolds created by indirect three-dimensional printing for tissue engineering. Biofabrication.

[37] Bose S, Vahabzadeh S, Bandyopadhyay A (2013). Bone tissue engineering using 3D printing. Mater Today.

[38] Zein I, Hutmacher DW, Tan KC, Teoh SH (2002). Fused deposition modeling of novel scaffold architectures for tissue engineering applications. Biomaterials.

[39] van Noort R (2012). The future of dental devices is digital. Dent Mater.

[40] Hutmacher DW, Schantz T, Zein I, Ng KW, Teoh SH, Tan KC (2001). Mechanical properties and cell cultural response of polycaprolactone scaffolds designed and fabricated via fused deposition modeling. J Biomed Mater Res.

[41] Park SH, Park DS, Shin JW, Kang YG, Kim HK, Yoon TR (2012). Scaffolds for bone tissue engineering fabricated from two different materials by the rapid prototyping technique: PCL versus PLGA. J Mater Sci Mater Med.

[42] Kim J, McBride S, Tellis B, Alvarez-Urena P, Song Y-H, Dean DD (2012). Rapid-prototyped PLGA/β-TCP/hydroxyapatite nanocomposite scaffolds in a rabbit femoral defect model. Biofabrication.

[43] Woodfield TB, Malda J, De Wijn J, Peters F, Riesle J, van Blitterswijk CA (2004). Design of porous scaffolds for cartilage tissue engineering using a three-dimensional fiber-deposition technique. Biomaterials.

[44] Kalita SJ, Bose S, Hosick HL, Bandyopadhyay A (2003). Development of controlled porosity polymer-ceramic composite scaffolds via fused deposition modeling. Mater Sci Eng C.

[45] Rai B, Teoh SH, Ho KH, Hutmacher DW, Cao T, Chen F (2004). The effect of rhBMP-2 on canine osteoblasts seeded onto 3D bioactive polycaprolactone scaffolds. Biomaterials.

[46] Korpela J, Kokkari A, Korhonen H, Malin M, Närhi T, Seppälä J (2013). Biodegradable and bioactive porous scaffold structures prepared using fused deposition modeling. J Biomed Mater Res B Appl Biomater.

[47] Yen H-J, Tseng C-S, S-h H, Tsai C-L (2009). Evaluation of chondrocyte growth in the highly porous scaffolds made by fused deposition manufacturing (FDM) filled with type II collagen. Biomed Microdevices.

[48] Teo EY, Ong S-Y, Khoon Chong MS, Zhang Z, Lu J, Moochhala S (2011). Polycaprolactone-based fused deposition modeled mesh for delivery of antibacterial agents to infected wounds. Biomaterials.

[49] Probst F, Hutmacher D, Müller D, Machens H, Schantz J (2010). Calvarial reconstruction by customized bioactive implant. Handchir Mikrochir Plast Chir.

[50] Shim J-H, Moon T-S, Yun M-J, Jeon Y-C, Jeong C-M, Cho D-W (2012). Stimulation of healing within a rabbit calvarial defect by a PCL/PLGA scaffold blended with TCP using solid freeform fabrication technology. J Mater Sci Mater Med.

[51] Kim JY, Cho D-W (2009). Blended PCL/PLGA scaffold fabrication using multi-head deposition system. Microelectron Eng.

[52] Van Bael S, Desmet T, Chai YC, Pyka G, Dubruel P, Kruth J-P (2013). In vitro cell-biological performance and structural characterization of selective laser sintered and plasma surface functionalized polycaprolactone scaffolds for bone regeneration. Mater Sci Eng C.

[53] Kang S-W, Bae J-H, Park S-A, Kim W-D, Park M-S, Ko Y-J (2012). Combination therapy with BMP-2 and BMSCs enhances bone healing efficacy of PCL scaffold fabricated using the 3D plotting system in a large segmental defect model. Biotechnol Lett.

[54] Shim J-H, Lee J-S, Kim JY, Cho D-W (2012). Bioprinting of a mechanically enhanced three-dimensional dual cell-laden construct for osteochondral tissue engineering using a multi-head tissue/organ building system. J Micromech Microeng.

[55] Espalin D, Arcaute K, Rodriguez D, Medina F, Posner M, Wicker R (2010). Fused deposition modeling of patient-specific polymethylmethacrylate implants. Rapid Prototyping J.

[56] Arburg. 3D printing with freeform from ARBURG.

[57] Dowler C (1989). Automatic model building cuts design time, costs. Plastics Eng.

[58] Fisher JP, Dean D, Mikos AG (2002). Photocrosslinking characteristics and mechanical properties of diethyl fumarate/poly (propylene fumarate) biomaterials. Biomaterials.

[59] Melchels FP, Feijen J, Grijpma DW (2010). A review on stereolithography and its applications in biomedical engineering. Biomaterials.

[60] Wang WL, Cheah CM, Fuh JYH, Lu L (1996). Influence of process parameters on stereolithography part shrinkage. Mater Design.

[61] Heller C, Schwentenwein M, Russmueller G, Varga F, Stampfl J, Liska R (2009). Vinyl esters: low cytotoxicity monomers for the fabrication of biocompatible 3D scaffolds by lithography based additive manufacturing. J Polym Sci A Polym Chem.

[62] Lee K-W, Wang S, Fox BC, Ritman EL, Yaszemski MJ, Lu L (2007). Poly (propylene fumarate) bone tissue engineering scaffold fabrication using stereolithography: effects of resin formulations and laser parameters. Biomacromolecules.

[63] Jansen J, Melchels FP, Grijpma DW, Feijen J (2008). Fumaric acid monoethyl ester-functionalized poly (d, l-lactide)/N-vinyl-2-pyrrolidone resins for the preparation of tissue engineering scaffolds by stereolithography. Biomacromolecules.

[64] Kang H-W, Cho D-W (2012). Development of an Indirect Stereolithography Technology for Scaffold Fabrication with a wide range of biomaterial selectivity. Tissue Eng Part C Methods.

[65] Park JH, Jung JW, Kang H-W, Cho D-W (2014). Indirect three-dimensional printing of synthetic polymer scaffold based on thermal molding process. Biofabrication.

[66] Zhang X, Jiang X, Sun C (1999). Micro-stereolithography of polymeric and ceramic microstructures. Sens Actuators A: Phys.

[67] Arcaute K, Mann B, Wicker R (2010). Stereolithography of spatially controlled multi-material bioactive poly (ethylene glycol) scaffolds. Acta Biomater.

[68] Choi J-W, Wicker R, Lee S-H, Choi K-H, Ha C-S, Chung I (2009). Fabrication of 3D biocompatible/biodegradable micro-scaffolds using dynamic mask projection microstereolithography. J Mater Process Technol.

[69] Elomaa L, Teixeira S, Hakala R, Korhonen H, Grijpma DW, Seppälä JV (2011). Preparation of poly (ε-caprolactone)-based tissue engineering scaffolds by stereolithography. Acta Biomater.

[70] Melchels FP, Feijen J, Grijpma DW (2009). A poly (D, L-lactide) resin for the preparation of tissue engineering scaffolds by stereolithography. Biomaterials.

[71] Seck TM, Melchels FP, Feijen J, Grijpma DW (2010). Designed biodegradable hydrogel structures prepared by stereolithography using poly (ethylene glycol)/poly (d, l-lactide)-based resins. J Control Release.

[72] Shin JH, Lee JW, Jung JH, Cho D-W, Lim G (2011). Evaluation of cell proliferation and differentiation on a poly (propylene fumarate) 3D scaffold treated with functional peptides. J Mater Sci.

[73] Kim K, Dean D, Wallace J, Breithaupt R, Mikos AG, Fisher JP (2011). The influence of stereolithographic scaffold architecture and composition on osteogenic signal expression with rat bone marrow stromal cells. Biomaterials.

[74] Lee JW, Kang KS, Lee SH, Kim J-Y, Lee B-K, Cho D-W (2011). Bone regeneration using a microstereolithography-produced customized poly (propylene fumarate)/diethyl fumarate photopolymer 3D scaffold incorporating BMP-2 loaded PLGA microspheres. Biomaterials.

[75] Lee JW, Ahn G, Kim DS, Cho D-W (2009). Development of nano-and microscale composite 3D scaffolds using PPF/DEF-HA and micro-stereolithography. Microelectron Eng.

[76] Schüller‐Ravoo S, Feijen J, Grijpma DW (2011). Preparation of flexible and elastic poly (trimethylene carbonate) structures by stereolithography. Macromol Biosci.

[77] Schüller‐Ravoo S, Teixeira SM, Feijen J, Grijpma DW, Poot AA (2013). Flexible and Elastic Scaffolds for Cartilage Tissue Engineering Prepared by Stereolithography Using Poly (trimethylene carbonate)‐Based Resins. Macromol Biosci.

[78] Gauvin R, Chen Y-C, Lee JW, Soman P, Zorlutuna P, Nichol JW (2012). Microfabrication of complex porous tissue engineering scaffolds using 3D projection stereolithography. Biomaterials.

[79] Kim K, Yeatts A, Dean D, Fisher JP (2010). Stereolithographic bone scaffold design parameters: osteogenic differentiation and signal expression. Tissue Eng Part B Rev.

[80] Melchels FP, Barradas A, Van Blitterswijk CA, De Boer J, Feijen J, Grijpma DW (2010). Effects of the architecture of tissue engineering scaffolds on cell seeding and culturing. Acta Biomater.

[81] Chan V, Zorlutuna P, Jeong JH, Kong H, Bashir R (2010). Three-dimensional photopatterning of hydrogels using stereolithography for long-term cell encapsulation. Lab Chip.

[82] Cui X, Breitenkamp K, Finn M, Lotz M, D'Lima DD (2012). Direct human cartilage repair using three-dimensional bioprinting technology. Tissue Eng Part A.

[83] Pattanayak DK, Fukuda A, Matsushita T, Takemoto M, Fujibayashi S, Sasaki K (2011). Bioactive Ti metal analogous to human cancellous bone: fabrication by selective laser melting and chemical treatments. Acta Biomater.

[84] Lohfeld S, Tyndyk M, Cahill S, Flaherty N, Barron V, McHugh P (2010). A method to fabricate small features on scaffolds for tissue engineering via selective laser sintering. J Biomed Sci Eng.

[85] Wiria FE, Leong KF, Chua CK, Liu Y (2007). Poly- < i > ε</i > -caprolactone/hydroxyapatite for tissue engineering scaffold fabrication via selective laser sintering. Acta Biomater.

[86] Tan K, Chua C, Leong K, Cheah C, Cheang P, Abu Bakar M (2003). Scaffold development using selective laser sintering of polyetheretherketone–hydroxyapatite biocomposite blends. Biomaterials.

[87] Yeong W, Sudarmadji N, Yu H, Chua C, Leong K, Venkatraman S (2010). Porous polycaprolactone scaffold for cardiac tissue engineering fabricated by selective laser sintering. Acta Biomater.

[88] Tan KH, Chua CK, Leong KF, Cheah CM, Gui WS, Tan WS (2005). Selective laser sintering of biocompatible polymers for applications in tissue engineering. Bio-Med Mater Eng.

[89] Chua C, Leong K, Tan K, Wiria F, Cheah C (2004). Development of tissue scaffolds using selective laser sintering of polyvinyl alcohol/hydroxyapatite biocomposite for craniofacial and joint defects. J Mater Sci Mater Med.

[90] Williams JM, Adewunmi A, Schek RM, Flanagan CL, Krebsbach PH, Feinberg SE (2005). Bone tissue engineering using polycaprolactone scaffolds fabricated via selective laser sintering. Biomaterials.

[91] Nickels L (2012). World's first patient-specific jaw implant. Metal Powder Report.

[92] Eshraghi S, Das S (2010). Mechanical and microstructural properties of polycaprolactone scaffolds with one-dimensional, two-dimensional, and three-dimensional orthogonally oriented porous architectures produced by selective laser sintering. Acta Biomater.

[93] Sudarmadji N, Tan J, Leong K, Chua C, Loh Y (2011). Investigation of the mechanical properties and porosity relationships in selective laser-sintered polyhedral for functionally graded scaffolds. Acta Biomater.

[94] Giannitelli S, Accoto D, Trombetta M, Rainer A (2014). Current trends in the design of scaffolds for computer-aided tissue engineering. Acta Biomater.

[95] Eshraghi S, Das S (2012). Micromechanical finite-element modeling and experimental characterization of the compressive mechanical properties of polycaprolactone–hydroxyapatite composite scaffolds prepared by selective laser sintering for bone tissue engineering. Acta Biomater.

[96] Eosoly S, Brabazon D, Lohfeld S, Looney L (2010). Selective laser sintering of hydroxyapatite/poly-ε-caprolactone scaffolds. Acta Biomater.

[97] Kang H, Hollister SJ, La Marca F, Park P, Lin C-Y (2013). Porous Biodegradable Lumbar Interbody Fusion Cage Design and Fabrication Using Integrated Global-Local Topology Optimization With Laser Sintering. J Biomech Eng.

[98] Liao HT, Lee MY, Tsai WW, Wang HC, Lu WC. Osteogenesis of adipose‐derived stem cells on polycaprolactone–β‐tricalcium phosphate scaffold fabricated via selective laser sintering and surface coating with collagen type I. J Tissue Eng Regenerative Med. 2013. 10.1002/term.1811 23955935

[99] Duan B, Wang M, Zhou WY, Cheung WL, Li ZY, Lu WW (2010). Three-dimensional nanocomposite scaffolds fabricated via selective laser sintering for bone tissue engineering. Acta Biomater.

[100] Duan B, Wang M (2010). Customized Ca–P/PHBV nanocomposite scaffolds for bone tissue engineering: design, fabrication, surface modification and sustained release of growth factor. J R Soc Interface.

[101] Shuai C, Mao Z, Lu H, Nie Y, Hu H, Peng S (2013). Fabrication of porous polyvinyl alcohol scaffold for bone tissue engineering via selective laser sintering. Biofabrication.

[102] Duan B, Wang M (2010). Encapsulation and release of biomolecules from Ca–P/PHBV nanocomposite microspheres and three-dimensional scaffolds fabricated by selective laser sintering. Polym Degrad Stab.

[103] Xia Y, Zhou P, Cheng X, Xie Y, Liang C, Li C (2013). Selective laser sintering fabrication of nano-hydroxyapatite/poly-ε-caprolactone scaffolds for bone tissue engineering applications. Int J Nanomedicine.

[104] C-j S, Z-z M, Z-k H, S-p P (2014). Preparation of complex porous scaffolds via selective laser sintering of poly (vinyl alcohol)/calcium silicate. Journal of Bioactive and Compatible Polymers: Biomedical Applications.

[105] Landers R, Mülhaupt R (2000). Desktop manufacturing of complex objects, prototypes and biomedical scaffolds by means of computer‐assisted design combined with computer‐guided 3D plotting of polymers and reactive oligomers. Macromol Mater Eng.

[106] Landers R, Hübner U, Schmelzeisen R, Mülhaupt R (2002). Rapid prototyping of scaffolds derived from thermoreversible hydrogels and tailored for applications in tissue engineering. Biomaterials.

[107] Maher P, Keatch R, Donnelly K, Paxton J. Formed 3D bio-scaffolds via rapid prototyping technology. 4th European Conference of the International Federation for Medical and Biological Engineering: Springer; 2009. p. 2200-4.

[108] Pataky K, Braschler T, Negro A, Renaud P, Lutolf MP, Brugger J (2012). Microdrop Printing of Hydrogel Bioinks into 3D Tissue‐Like Geometries. Adv Mater.

[109] Haberstroh K, Ritter K, Kuschnierz J, Bormann KH, Kaps C, Carvalho C (2010). Bone repair by cell‐seeded 3D‐bioplotted composite scaffolds made of collagen treated tricalciumphosphate or tricalciumphosphate‐chitosan‐collagen hydrogel or PLGA in ovine critical‐sized calvarial defects. J Biomed Mater Res B Appl Biomater.

[110] Cohen DL, Malone E, Lipson H, Bonassar LJ (2006). Direct freeform fabrication of seeded hydrogels in arbitrary geometries. Tissue Eng.

[111] Nakamura M, Kobayashi A, Takagi F, Watanabe A, Hiruma Y, Ohuchi K (2005). Biocompatible inkjet printing technique for designed seeding of individual living cells. Tissue Eng.

[112] Odde DJ, Renn MJ (2000). Laser‐guided direct writing of living cells. Biotechnol Bioeng.

[113] Koch L, Kuhn S, Sorg H, Gruene M, Schlie S, Gaebel R (2009). Laser printing of skin cells and human stem cells. Tissue Eng Part C Methods.

[114] Moon S, Hasan SK, Song YS, Xu F, Keles HO, Manzur F (2009). Layer by layer three-dimensional tissue epitaxy by cell-laden hydrogel droplets. Tissue Eng Part C Methods.

[115] Lim TC, Chian KS, Leong KF (2010). Cryogenic prototyping of chitosan scaffolds with controlled micro and macro architecture and their effect on in vivo neo‐vascularization and cellular infiltration. J Biomed Mater Res A.

[116] Lee H, Kim Y, Kim S, Kim G. Mineralized biomimetic collagen/alginate/silica composite scaffolds fabricated by a low-temperature bio-plotting process for hard tissue regeneration: fabrication, characterisation and in vitro cellular activities. J Mater Chem B. 2014. 10.1039/c4tb00931b 32262022

[117] Chien KB, Makridakis E, Shah RN (2012). Three-dimensional printing of soy protein scaffolds for tissue regeneration. Tissue Eng Part C Methods.

[118] Chien KB, Aguado BA, Bryce PJ, Shah RN (2013). In vivo acute and humoral response to three-dimensional porous soy protein scaffolds. Acta Biomater.

[119] Norotte C, Marga FS, Niklason LE, Forgacs G (2009). Scaffold-free vascular tissue engineering using bioprinting. Biomaterials.

[120] Skardal A, Zhang J, Prestwich GD (2010). Bioprinting vessel-like constructs using hyaluronan hydrogels crosslinked with tetrahedral polyethylene glycol tetracrylates. Biomaterials.

[121] Cui X, Boland T (2009). Human microvasculature fabrication using thermal inkjet printing technology. Biomaterials.

[122] Zhang K, Chou C-K, Xia X, Hung M-C, Qin L. Block-Cell-Printing for live single-cell printing. Proceedings of the National Academy of Sciences. 2014:201313661 10.1073/pnas.1313661111 PMC3939871 24516129

[123] Ahn S, Lee H, Lee EJ, Kim G (2014). A direct cell printing supplemented with low-temperature processing method for obtaining highly porous three-dimensional cell-laden scaffolds. J Mater Chem B.

[124] Kundu J, Shim JH, Jang J, Kim SW, Cho DW. An additive manufacturing‐based PCL–alginate–chondrocyte bioprinted scaffold for cartilage tissue engineering. J Tissue Eng Regenerative Med. 2013. 10.1002/term.1682 23349081

[125] Lee J-S, Hong JM, Jung JW, Shim J-H, Oh J-H, Cho D-W (2014). 3D printing of composite tissue with complex shape applied to ear regeneration. Biofabrication.

[126] Pati F, Jang J, Ha D-H, Kim SW, Rhie J-W, Shim J-H, et al. Printing three-dimensional tissue analogues with decellularized extracellular matrix bioink. Nat Commun. 2014;5 10.1038/ncomms4935 PMC4059935 24887553

[127] Xu T, Jin J, Gregory C, Hickman JJ, Boland T (2005). Inkjet printing of viable mammalian cells. Biomaterials.

[128] Dunn JCY, Chan WY, Cristini V, Kim JS, Lowengrub J, Singh S (2006). Analysis of cell growth in three-dimensional scaffolds. Tissue Eng.

